# SARS-CoV-2 Omicron variant BA.2 neutralisation in sera of people with Comirnaty or CoronaVac vaccination, infection or breakthrough infection, Hong Kong, 2020 to 2022

**DOI:** 10.2807/1560-7917.ES.2022.27.18.2200178

**Published:** 2022-05-05

**Authors:** Samuel MS Cheng, Chris Ka Pun Mok, Karl CK Chan, Susanna S Ng, Bosco HS Lam, Leo LH Luk, Fanny W Ko, Chunke Chen, Karen Yiu, John KC Li, Ken KP Chan, Leo CH Tsang, Leo LM Poon, David SC Hui, Malik Peiris

**Affiliations:** 1 School of Public Health, LKS Faculty of Medicine, The University of Hong Kong, Hong Kong SAR, China; 2 These authors contributed equally to the research; 3 The Jockey Club School of Public Health and Primary Care, The Chinese University of Hong Kong, Hong Kong SAR, China; 4 Li Ka Shing Institute of Health Sciences, Faculty of Medicine, The Chinese University of Hong Kong, Hong Kong SAR, China.; 5 Department of Medicine & Therapeutics, Faculty of Medicine, The Chinese University of Hong Kong, Hong Kong SAR, China; 6 Department of Pathology, North Lantau Hospital, Hong Kong SAR, China; 7 Centre for Immunology and Infection, Hong Kong Science Park, Shatin, Hong Kong SAR, China; 8 Stanley Ho Centre for Emerging Infectious Diseases, Faculty of Medicine, The Chinese University of Hong Kong, Hong Kong SAR, China

**Keywords:** SARS-CoV-2, Omicron, subvariant BA.2, subvariant BA.1, neutralization, vaccine, BNT162b2, CoronaVac, hybrid-immunity

## Abstract

**Background:**

Omicron subvariant BA.2 circulation is rapidly increasing globally.

**Aim:**

We evaluated the neutralising antibody response from vaccination or prior SARS-CoV-2 infection against symptomatic infection by BA.2 or other variants.

**Methods:**

Using 50% plaque reduction neutralisation tests (PRNT_50_), we assessed neutralising antibody titres to BA.2, wild type (WT) SARS-CoV-2 and other variants in Comirnaty or CoronaVac vaccinees, with or without prior WT-SARS-CoV-2 infection. Titres were also measured for non-vaccinees convalescing from a WT-SARS-CoV-2 infection. Neutralising antibodies in BA.2 and BA.1 breakthrough infections and in BA.2 infections affecting non-vaccinees were additionally studied.

**Results:**

In vaccinees or prior WT-SARS-CoV-2-infected people, BA.2 and BA.1 PRNT_50_ titres were comparable but significantly (p < 10 ^− 5^) lower than WT. In each group of 20 vaccinees with (i) three-doses of Comirnaty, (ii) two CoronaVac followed by one Comirnaty dose, or (iii) one dose of either vaccine after a WT-SARS-CoV-2 infection, ≥ 19 individuals developed detectable (PRNT_50_ titre ≥ 10) antibodies to BA.2, while only 15 of 20 vaccinated with three doses of CoronaVac did. Comirnaty vaccination elicited higher titres to BA.2 than CoronaVac. In people convalescing from a WT-SARS-CoV-2 infection, a single vaccine dose induced higher BA.2 titres than three Comirnaty (p = 0.02) or CoronaVac (p = 0.00001) doses in infection-naïve individuals. BA.2 infections in previously uninfected and unvaccinated individuals elicited low (PRNT_50_ titre ≤ 80) responses with little cross-neutralisation of other variants. However, vaccinees with BA.1 or BA.2 breakthrough infections had broad cross-neutralising antibodies to WT viruses, and BA.1, BA.2, Beta and Delta variants.

**Conclusions:**

Existing vaccines can be of help against the BA.2 subvariant.

## Introduction

A new variant of severe acute respiratory syndrome coronavirus 2 (SARS-CoV-2), the virus that causes coronavirus disease (COVID-19) emerged in South Africa in November 2021 [1]. This variant within Phylogenetic Assignment of Named Global Outbreak (Pango) lineage B.1.1.529 was designated as a ‘variant of concern’ and named Omicron [1]. It had 37 amino-acid changes in the virus spike protein compared with the wild-type (WT) virus and appeared more transmissible than all previously identified virus variants [2,3]. Early assessments suggested, however, that it might be associated with reduced disease severity [4]. Up to the end of 2021, three subvariants of Omicron were initially identified, namely BA.1, BA.2 and BA.3, with BA.1 being the first to spread worldwide [2].

An RNA vaccine (Comirnaty, BNT162b2, BioNTech-Pfizer, Mainz, Germany/New York, United States) and an inactivated whole-virus vaccine (CoronaVac, Sinovac Biotech Ltd, Beijing, China) are two of the most widely used COVID-19 vaccines globally, each having had over 2 billion doses delivered so far [5]. We and others have shown that Omicron BA.1 is poorly neutralised by sera from individuals vaccinated with two doses of Comirnaty or CoronaVac respectively [6,7]. In those previously vaccinated with two doses of CoronaVac, an additional dose of Comirnaty increased BA.1 neutralising antibody titres to higher levels than an additional dose of CoronaVac. Vaccine effectiveness studies have shown marked reduction of protection against symptomatic Omicron infection from two doses of RNA vaccines but improved protection associated with a third vaccine dose [8]. The reduction in vaccine protection prompted the development of Omicron BA.1-specific vaccines which are currently under evaluation [9].

More recently, circulation of Omicron subvariant BA.2 has been increasing in a number of countries, and this subvariant appears to have an even higher transmissibility than BA.1 [10]. Although BA.1 and BA.2 share 21 amino-acid changes in the spike protein relative to WT virus, they differ from each other by around 26 amino-acid residues, some of these being in the receptor binding (RBD) and N-terminal domains (NTD) [2]. Thus, it is possible that there are antigenic differences between BA.1 and BA.2. It is of public health importance to assess how well existing vaccines protect against BA.2. Neutralising antibodies are the best available correlate of protection [11]. Therefore, investigating how vaccine-immune sera neutralise BA.2 will provide an assessment of likely protection from existing vaccines, vaccine combinations and ‘hybrid immunity’ (i.e. immunity following both natural infection and vaccination) against BA.2 [12]. The aim of the present study was to assess plaque reduction neutralisation test (PRNT) antibody titres to BA.2 and compare them with WT and BA.1, in cohorts of infection-naïve individuals vaccinated with Comirnaty or CoronaVac vaccines and in those convalescing from WT SARS-CoV-2 infections with or without vaccination. We also compared neutralising antibody titres to BA.1, BA.2, WT, Delta and Beta variant viruses in acute and convalescent sera of individuals with vaccine-breakthrough BA.1 or BA.2 infections, as well as in sera of non-vaccinated people who had just had BA.2 infections.

## Methods

### Study design and clinical specimens

We randomly selected a subset of 20 individuals from each of six cohorts used in a previous study comparing PRNT antibody titres to WT and BA.1 subvariant of Omicron [6]. These included infection naïve individuals vaccinated with three doses of Comirnaty or CoronaVac vaccines or those vaccinated with two doses of CoronaVac and a subsequent dose of Comirnaty. Previously unvaccinated individuals with a past WT-SARS-CoV-2 infection (143–196 days post infection) were selected to represent waning antibody titres in convalescence. Sera from individuals with past SARS-CoV-2 infections vaccinated with one dose of Comirnaty or CoronaVac were included to investigate ‘hybrid immunity’. In vaccinated individuals, serum was collected 3–5 weeks after the last dose of vaccine. This study was carried out during the period from 21 February 2020 to 20 November 2021. Some of the patient cohorts followed in the current work have been included in previous studies of antibody and T-cell responses to SARS-CoV-2 [13,14].

Separately, between 20 November 2021 and 21 February 2022, convalescent (where possible, acute) sera were collected from patients diagnosed in Hong Kong with Omicron BA.1 (n = 14) and presumed BA.2 (n = 27) variant infection. All BA.1 infections were confirmed by sequencing and the presumed BA.2 infections were either confirmed by sequencing or occurred after 21 January 2022, a period when ≥ 97% sequenced infections in Hong Kong were BA.2 (data not shown). All the BA.1-infected, and 20 of the BA.2-infected individuals had vaccine breakthrough infections. Of the total 34 people with breakthrough infection, 22 were vaccinated with Comirnaty and nine with CoronaVac. One further individual had received Vaxzevria (ChAdOx1/nCoV-19, AstraZeneca, Cambridge, United Kingdom), another Spikevax (mRNA-1273, Moderna, Cambridge, United States) and one both Comirnaty and COVID-19 Vaccine Janssen (Janssen-Cilag International NV, Beerse, Belgium).

### Virus strains

We isolated a Pango lineage B.1.1.529 (Omicron) subvariant BA.2 strain (hCoV-19/Hong Kong/VM22000135_HKUVOC0588P2/2022) from a patient with COVID-19 in Hong Kong in Vero-E6 cells over-expressing TMPRSS2 [15]. The amino-acid sequence (GISAID EPI_ISL_9570707) of the virus isolate was identical to the virus in the original clinical specimen except at one position. This was due to a non-synonymous substitution in the nt sequence at position C11454T in open reading frame (ORF)1a.

A Pango lineage B.1.1.529 (Omicron) subvariant BA.1 virus hCoV-19/Hong Kong/VM21044713_WHP5047-S5/2021 and a WT SARS-CoV-2 Pango lineage A virus BetaCoV/Hong Kong/VM20001061/2020 (WT) were used for comparison [6]. The sequences of these viruses are available in GISAID as EPI_ISL_6716902 and EPI_ISL_412028 respectively.

### Plaque reduction neutralisation tests

The PRNT assays were carried out in duplicate using 24-well tissue culture plates (TPP Techno Plastic Products AG, Trasadingen, Switzerland) in a biosafety level 3 facility using Vero E6 TMPRSS2 cells [15] as previously described and validated [16]. All sera were heat-inactivated at 56 °C for 30 min before testing. Serial twofold dilutions from 1:10 to 1:320 of each serum sample were incubated with 30–40 plaque-forming units of virus for 1 hour at 37 °C and then the virus–serum mixtures were added onto pre-formed cell monolayers and incubated for 1 hour at 37 °C in a 5% CO_2_ incubator. The cell monolayer was then overlaid with 1% agarose in cell culture medium and incubated for 3 days, at which time the plates were fixed and stained. Antibody titres were defined as the highest serum dilution that resulted in ≥ 50% (PRNT_50_) reduction in the number of virus plaques. The mean of the plaque numbers observed in the duplicate dilution-series was used for this computation. Virus back titrations, positive and negative control sera were included in every experiment.

### Surrogate virus neutralisation tests

The surrogate virus neutralisation test (sVNT) was performed according to the manufacturer’s recommendations (Genescript, New Jersey, United States) using SARS-CoV-2 spike RBD to WT and virus variants Beta and Delta. Inhibition (%) of binding of the RBD to angiotensin converting enzyme-2 (ACE2) of ≥ 30% was regarded as positive.

### Statistical analysis

For sample size calculations, the maximum standard deviation (SD) of log PRNT_50_ antibody titres for the uninfected vaccinated groups was 1.37. Assuming a threefold difference in geometric mean of PRNT_50_ antibody titre (GMT), a sample size of 10 in each group would have statistical power of > 0.99 for detecting a difference between groups using the two-tailed Mann–Whitney U test. Comparisons between groups with larger sample size or smaller within-group variation would have larger statistical power.

Categorical variables were summarised as proportions or percentage and continuous variables were summarised as geometric mean with SD. Sera with undetectable (< 10) antibody titres were assigned an antibody titre of 5, for purposes of GMT calculations or statistical comparisons. GMTs were designated as reciprocal of the serum dilution throughout the text. Differences in antibody titres to WT, BA.1 and BA.2 viruses were assessed using the Wilcoxon signed-rank test when comparing paired antibody titres to different viruses in the same serum and the two-tailed Mann–Whitney U test when comparing different groups of individuals. Absolute p values were provided. p values < 0.05 were considered statistically significant.

## Results

### Antibody titres to BA.2, BA.1 and wild-type virus in vaccinated individuals and in convalescent individuals with or without immunisation

Sera were tested to determine PRNT_50_ antibody titres to Omicron subvariant BA.2 and the data compared with previously reported data on those sera for the WT or Omicron BA.1 viruses [6]. The age and sex (collected as a binary variable) demographics, GMT (95% confidence interval (CI)) for each virus for each cohort and the number (and percentage) of sera with detectable antibody are shown in Table 1. 

**Table 1:** Age, sex, geometric mean of PRNT_50_ antibody titres to BA.2, BA.1 and WT virus at 3–5 weeks post last dose of vaccine, Hong Kong, 21 February 2020–20 November 2021 (n = 120)

Exposure group	Number	Mean age (SD) in years	Age range in years	M: F	WT GMT (95% CI)	BA.1 GMT (95% CI)	BA.2 GMT (95% CI)	Number with BA.2 PRNT_50_ titres above indicated thresholds	
								≥ 1:10^a^	≥ 1:25.6^b^
Comirnaty (3 doses)	20	49.5 (14.8)	22–72	11:9	320.0 (320.0–320.0)	67.3 (45.5–99.6)	95.1 (73.7–122.8)	20	20
CoronaVac (3 doses)	20	49.8 (7.9)	36–68	4:16	65.0 (43.8–96.5)	8.1 (6.4–10.3)	9.3 (7.8–11.5)	15	0
CoronaVac (2 doses) + Comirnaty (1 dose)	20	48.4 (9.2)	31–66	10:10	309.1 (287.5–332.4)	51.0 (32.9–79.0)	46.0 (29.2–72.3)	19	16
SARS-CoV-2 (WT) convalescent	20	48.7 (15.0)	20–70	7:13	82.8 (58.0–118.3)	8.1 (5.5–12.1)	9.0 (6.7–12.2)	11	1
SARS-CoV-2 (WT) convalescent + Comirnaty (1 dose)	20	48.9 (15.2)	20–70	8:12	320.0 (320.0–320.0)	121.3 (85.0–173.0)	144.2 (113.2–183.6)	20	20
SARS-CoV-2 (WT) convalescent + CoronaVac (1 dose)	20	55.9 (10.0)	37–81	12:8	226.3 (185.8–275.5)	31.4 (22.8–43.2)	28.3 (20.8–38.4)	20	10

CI: confidence interval; F: female; GMT: geometric mean of PRNT_50_ antibody titre; M: male; PRNT_50_: 50% plaque reduction neutralisation test; SARS-CoV-2: severe acute respiratory syndrome coronavirus 2; SD: standard deviation; WT: wild type.

^a^ ≥ 1:10 is the threshold for detectable antibody titres.

^b^ ≥ 1:25.6 is an antibody titre threshold, predictive of conferring 50% protection against SARS-CoV-2 infection [11,16].

**Figure 1:**
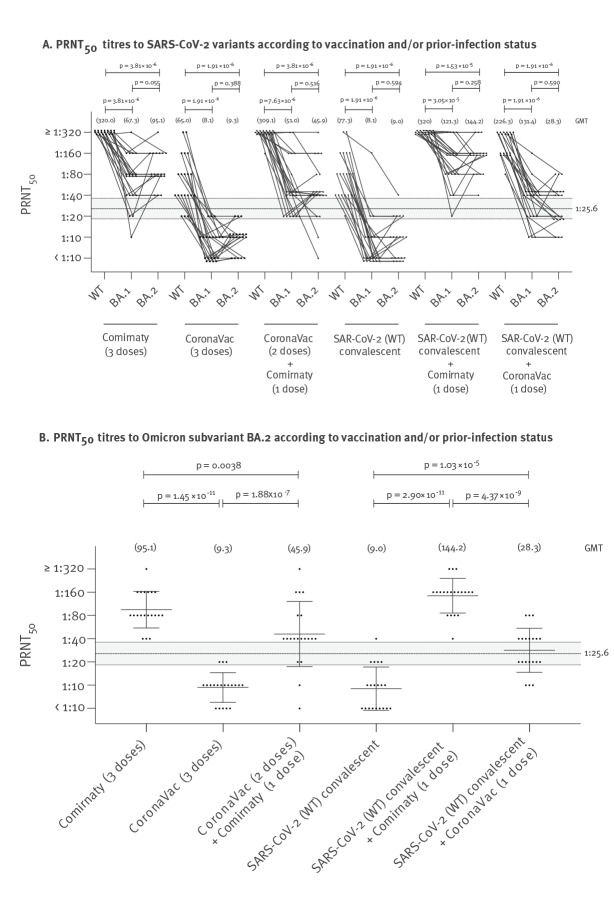
PRNT_50_ antibody titres to WT SARS-CoV-2 and Omicron subvariants BA.1 and BA.2, in infection-naïve and previously-infected (by WT virus) individuals, according to Comirnaty or CoronaVac vaccination, Hong Kong, 21 February–20 November 2021 (n = 120)

GMT: geometric mean of neutralising antibody titres; PRNT_50_ : 50% plaque reduction neutralisation test; SARS-CoV-2: severe acute respiratory syndrome coronavirus 2; SD: standard deviation; WT: wild type.

The PRNT_50_ antibody titres and GMT to BA.2 were not significantly different from those to BA.1 but both subvariants’ GMTs were significantly lower (p < 10 ^− 5^) than GMTs to WT virus (Figure 1). Nineteen or more individuals in each of the 20-member cohorts vaccinated with three doses of Comirnaty, two doses of CoronaVac followed by one dose of Comirnaty, or of SARS-CoV-2 convalescent individuals who had been given one dose of either Comirnaty or CoronaVac vaccine had detectable (≥ 10) PRNT_50_ antibody to BA.2 with GMTs of 95.1, 46.0, 144.2 or 28.3, respectively (Table 1). Fifteen of 20 who were given three doses of CoronaVac had detectable PRNT_50_ antibody albeit with lower titres (GMT: 9.3). Interestingly, those with past infection with the WT SARS-CoV-2, who were given one dose of Comirnaty had significantly higher PRNT_50_ antibody titres to BA.2 (GMT: 144.2) compared with those with three doses of Comirnaty (GMT: 95.1; p = 0.02 in two-tailed Mann–Whitney U test). Similarly, those with past infection given one dose of CoronaVac had significantly higher BA.2 PRNT_50_ titres (GMT: 28.3) compared with those given three doses of CoronaVac (GMT: 9.3; p = 0.00001).

A. PRNT_50_ antibody titres of each individual are linked by a line. GMTs for each group are indicated above in parentheses. Antibody titres between pairs of viruses were compared using the Wilcoxon signed-rank test.

B. PRNT_50_ antibody titres to Omicron subvariant BA.2 in the different groups, with geometric mean +/− SD presented for ease of comparison (Table 1 displays the numbers of individuals in each group). The dashed line indicates the predicted threshold of PRNT_50_ antibody titre (1:25.6) conferring 50% protection from infection, and shading indicates 95% confidence intervals [11,16]. Mann–Whitney test (two tailed) was used for significance testing. Exact p values are denoted.

There is as yet no universally agreed correlate of protection for SARS-CoV-2 infection. However, Khoury and colleagues previously used data from multiple vaccine efficacy trials to establish a predicted neutralising antibody threshold associated with 50% protection from infection to be 20.2% (95% CI: 14.4–28.4) of the GMT in convalescent individuals [11]. These predicted thresholds were also validated against protection of vaccinated individuals against WT virus and against variants of concern [17]. While these predicted thresholds of protection are not definitive, they may have some utility in assessing immune status following natural infection or vaccination. Using our panel of SARS-CoV-2 convalescent sera collected 30–60 days post infection, we previously estimated this predicted threshold in our PRNT_50_ assay to be a titre of 25.6 (95% CI: 18.3–36.0) [16]. We used this PRNT_50_ threshold as a guide to assess adequacy of PRNT responses. Applying this predicted 50% protective threshold to our current PRNT_50_ titres with BA.2, we estimate that all of 20 individuals with three doses Comirnaty and all of SARS-CoV-2 convalescent individuals with one dose of Comirnaty meet this predicted threshold, as well as 16 of 20 people vaccinated with two doses of CoronaVac followed by one dose of Comirnaty and 10 of 20 SARS-CoV-2 convalescent individuals with one dose of CoronaVac. In contrast, none of the 20 individuals with three doses of CoronaVac and only one of 20 people convalescing from SARS-CoV-2 without any vaccine did so (Table 1, Figure 1B).

**Figure 2:**
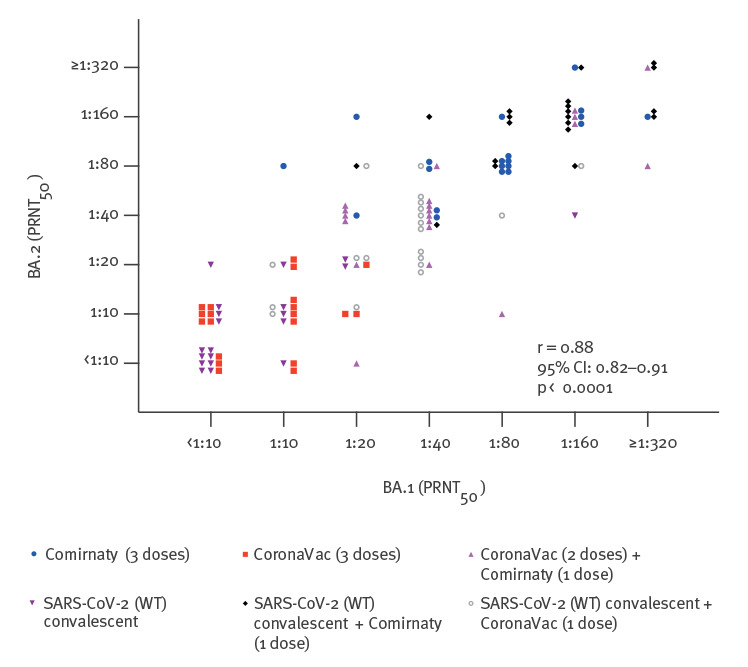
Correlation of Omicron subvariant BA.2 and BA.1 PRNT_50_ antibody titres, Hong Kong, 21 February 2020–20 November 2021 (n = 120)

CI: confidence interval; PRNT_50_: 50% plaque reduction neutralisation test; r: Spearman correlation coefficient; SARS-CoV-2: severe acute respiratory syndrome coronavirus 2; WT: wild type.

Taking the 120 individuals in the six cohorts investigated together, it was notable that the PRNT_50_ titres to BA.1 and BA.2 were well correlated with each other (Spearman correlation coefficient r = 0.88; 95% CI: 0.82–0.91; p < 0.0001) with titres generally being within fourfold dilutions of each other (Figure 2).

### Neutralising antibodies to SARS-CoV-2 variants in convalescent/acute sera from BA.1- or BA.2-infected individuals with or without prior vaccination 

Convalescent (where possible, acute) sera were obtained from 14 BA.1-infected individuals (all with breakthrough infections) and from 27 presumed BA.2-infected individuals (including 20 with breakthrough infection). The demographics, vaccine history and timing of first reverse transcription-PCR (in asymptomatic infections) or onset of symptoms, before collection of serum are shown in Table 2. 

**Table 2:** PRNT_50_ titres in BA.1 and BA.2 infections in vaccinated or unvaccinated individuals, Hong Kong, 13 November 2021–10 February 2022 (n = 41)

Case	Age group (years), sex	Severity	Vaccine	Number of doses	Day^a^ of 1^st^ serum collection	Day^a^ of 2^nd^ serum collection	BA.1 PRNT_50_		BA.2 PRNT_50_		WT PRNT_50_	
							Acute	Convalescent	Acute	Convalescent	Acute	Convalescent
**BA.1 breakthrough infections in vaccinated cases**												
1	31–40, M	Mild	Comirnaty	2	1	14	< 10	≥ 320	< 10	160	80	≥ 320
2	31–40, M	Asymptomatic	Spikevax	2	1	5	< 10	40	< 10	NA	160	≥ 320
3	31–40, M	Mild	Comirnaty	2	0	7	10	160	< 10	NA	20	≥ 320
4	21–30, M	Mild	Comirnaty	2	1	8	40	≥ 320	20	160	160	≥ 320
5	41–50, M	Asymptomatic	Comirnaty	2	1	17	10	80	< 10	80	20	160
6	21–30, M	Mild	Comirnaty	2	1	7	10	80	< 10	80	160	≥ 320
7	41–50, M	Asymptomatic	Vaxzevria	2	2	14	< 10	160	< 10	40	< 10	≥ 320
8	21–30, F	Mild	CoronaVac	2	0	7	< 10	40	NA	40	10	≥ 320
9	11–20, F	Asymptomatic	Janssen/Comirnaty	2	1	14	20	≥ 320	20	≥ 320	20	≥ 320
10	31–40, M	Mild	Comirnaty	2	1	13	10	160	10	≥ 320	20	≥ 320
11	21–30, F	Asymptomatic	Comirnaty	2	1	15	20	≥ 320	10	160	< 10	160
12	31–40, M	Asymptomatic	Comirnaty	3	1	25	20	≥ 320	20	≥ 320	80	≥ 320
13	71–80, F	Mild	Comirnaty	3	2	13	40	≥ 320	40	≥ 320	80	160
14	61–70, M	Mild	Comirnaty	2	NA	19	NA	≥ 320	NA	≥ 320	NA	≥ 320
**GMT** **(95%** **CI)**							**11.7** **(7.4–18.5)**	**168.1** **(106.5–265.4)**	**9.4** **(5.9–15.2)**	**151.0** **(90.4–252.2)**	**34.1** **(15.8–73.5)**	**275.8** **(232.6–327.1)**
**BA.2 breakthrough infections in vaccinated cases**												
15	31–40, F	Asymptomatic	CoronaVac	2	1	NA	20	NA	40	NA	80	NA
16	41–50, M	Mild	Comirnaty	2	2	16	10	≥ 320	< 10	≥ 320	40	≥ 320
17	61–70, F	Moderate	Comirnaty	3	2	14	40	80	40	160	≥ 320	≥ 320
18	31–40, F	Mild	Comirnaty	2	3	32	< 10	160	< 10	160	40	≥ 320
19	61–70, M	Mild	CoronaVac	3	3	39	10	40	< 10	80	10	≥ 320
20	51–60, M	Mild	Comirnaty	2	3	32	< 10	≥ 320	< 10	≥ 320	40	≥ 320
21	21–30, F	Mild	CoronaVac	2	1	29	< 10	≥ 320	< 10	≥ 320	10	≥ 320
22	41–50, M	Mild	Comirnaty	1	3	29	10	10	< 10	20	10	10
23	31–40, F	Mild	Comirnaty	2	2	35	< 10	≥ 320	< 10	≥ 320	40	≥ 320
24	31–40, F	Mild	Comirnaty	2	4	9	10	≥ 320	10	≥ 320	≥ 320	≥ 320
25	61–70, M	Mild	CoronaVac	2	3	46	< 10	20	< 10	20	< 10	≥ 320
26	31–40, M	Mild	Comirnaty	2	2	52	< 10	≥ 320	10	≥ 320	20	≥ 320
27	21–30, F	Mild	Comirnaty	2	1	37	10	≥ 320	< 10	≥ 320	20	≥ 320
28	21–30, F	Mild	Comirnaty	2	2	38	< 10	80	10	80	40	≥ 320
29	61–70, M	Asymptomatic	CoronaVac	1	1	51	10	10	< 10	20	< 10	40
30	41–50, F	Mild	CoronaVac	3	3	46	10	160	< 10	80	80	≥ 320
31	61–70, F	Mild	CoronaVac	2	5	48	< 10	160	< 10	≥ 320	10	≥ 320
32	71–80, M	Mild	Comirnaty	1	5	10	< 10	< 10	< 10	10	10	40
33	71–80, M	Asymptomatic	CoronaVac	2	5	30	10	10	20	40	20	20
34	41–50, M	Mild	Comirnaty	1	5	48	< 10	20	< 10	80	80	160
**GMT** **(95%** **CI)**							**7.8** **(6.0–10.2)**	**77.1** **(37.7–158.0)**	**7.3** **(5.3–10.1)**	**107.1** **(60.5–189.6)**	**28.3** **(16.1–49.6)**	**178.5** **(104.3–305.4)**
**BA.2 infections in unvaccinated cases^b^**												
35	51–60, M	Mild	Unvaccinated	0	5	18	< 10	20	< 10	80	< 10	10
36	61–70, M	Asymptomatic	Unvaccinated	0	0	32	< 10	10	< 10	40	< 10	< 10
37	81–90, M	Mild	Unvaccinated	0	2	45	< 10	40	< 10	80	< 10	10
38	61–70, F	Mild	Unvaccinated	0	4	7	< 10	< 10	< 10	< 10	< 10	< 10
39	61–70, F	Mild	Unvaccinated	0	2	52	< 10	< 10	< 10	20	< 10	< 10
40	61–70, M	Asymptomatic	Unvaccinated	0	3	44	< 10	< 10	< 10	20	< 10	10
41	71–80, F	Mild	Unvaccinated	0	3	23	< 10	< 10	< 10	10	< 10	< 10
**GMT** **(95%** **CI)**							**5.0** **(5.0–5.0)**	**9.1** **(4.2–19.7)**	**5.0** **(5.0–5.0)**	**24.4** **(9.3–63.6)**	**5.0** **(5.0–5.0)**	**6.7** **(4.8–9.5)**

CI: confidence interval; COVID-19: coronavirus disease; F: female; GMT: geometric mean of PRNT_50_ antibody titre; M: male; Janssen: COVID-19 Vaccine Janssen; NA: not available, because insufficient serum available for testing; PRNT_50_: 50% plaque reduction neutralisation test; RT-PCR: reverse transcription PCR; SARS-CoV-2: severe acute respiratory syndrome coronavirus 2; SD: standard deviation; WT: wild type.

^a^ Number of days elapsed between the time of symptom onset or the first RT-PCR positive test (for asymptomatic infection) and serum collection.

^b^ These cases were not known to have been previously infected with SARS-CoV-2.

**Figure 3:**
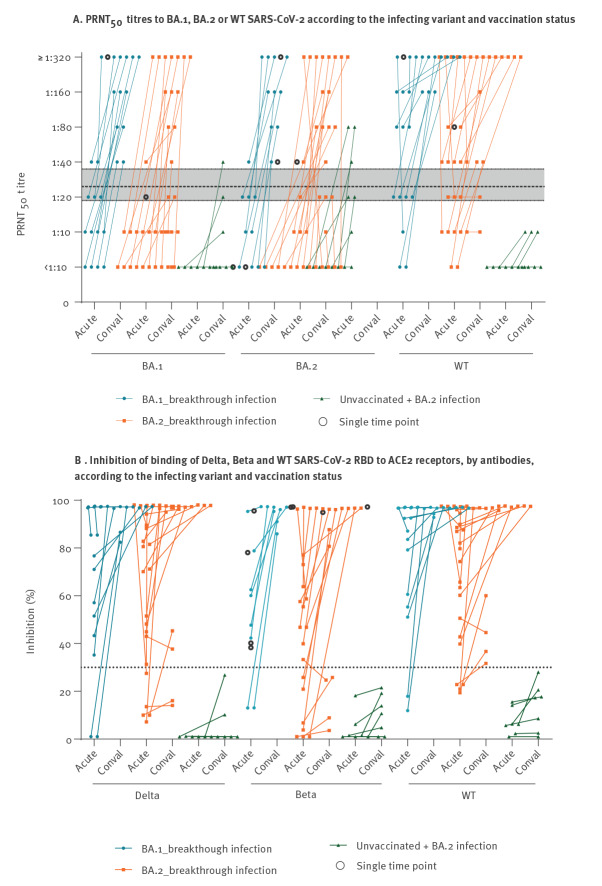
Serum neutralising antibody activity against WT and SARS-CoV-2 variants according to the virus variant causing infection and vaccination status, Hong Kong, 13 November 2021–10 February 2022 (n = 41)

ACE2: angiotensin converting enzyme-2; conval: convalescent; PRNT_50_: 50% plaque reduction neutralisation test; RBD: receptor binding domain; SARS-CoV-2: severe acute respiratory syndrome coronavirus 2; WT: wild type.

In previously vaccinated individuals, BA.1 breakthrough infections led to PRNT_50_ GMT to BA.1, BA.2 and WT increasing by 14.4, 16.1 and 8.1-fold, respectively (Table 2, Figure 3A). Similarly, in BA.2 breakthrough infections PRNT_50_ GMT to BA.1, BA.2 and WT increased by 9.9, 14.7 and 6.3-fold respectively. The mean per cent antibody-mediated inhibition of ACE2 binding to the spike receptor binding domains of Delta and Beta increased from < 55% to > 70% in BA.1 and BA.2 breakthrough infections (Figure 3B). In marked contrast, convalescent sera in those without prior vaccination infected with BA.2 virus had relatively low PRNT_50_ (GMT: 24.4) to the infecting BA.2 virus and even lower PRNT_50_ to BA.1 (GMT: 9.1) and WT (GMT: 6.7), respectively.

A. PRNT_50_ antibody titres to BA.1, BA.2 or WT viruses in acute and convalescent sera of previously uninfected people with BA.1 (n = 14) and BA.2 (n = 20) breakthrough infections as well as in previously uninfected unvaccinated individuals (n = 7) with BA.2 infections (Table 2 displays details on age, sex, type of vaccine, days after onset of infection). Black open circles indicate data from single acute or convalescent sera with no paired sera. The dashed horizontal line indicates the predicted threshold of PRNT_50_ antibody titre conferring 50% protection (25.6), and shading indicates 95% confidence intervals [11,16].

B. Surrogate virus neutralisation test (sVNT): antibody activity against Delta, Beta and WT is assessed as % inhibition of binding of these viruses’ RBD to SARS-CoV-2 ACE2 receptors. Positive cut off was 30% inhibition, as indicated by the dashed horizontal line.

## Discussion

In all cohorts of vaccinated individuals and those convalescent from WT-SARS-CoV-2 infection with or without vaccination, we found that that PRNT_50_ titres to BA.2 were markedly and significantly lower than those for WT virus, but that PRNT_50_ titres to BA.1 and BA.2 were comparable with each other, generally within fourfold dilution-steps of each other. These findings were broadly concordant with a recent report using pseudoparticle neutralisation assays where neutralisation titres to BA.1 and BA.2 viruses were markedly lower than to WT virus in Spikevax and Comirnaty vaccinated individuals and the BA.1 and BA.2 titres were comparable with each other in vaccine sera [18].

The spike proteins of our BA.1 and BA.2 virus isolates have the mutations previously reported [2], with BA.1 and BA.2, respectively, having 26 and 24 amino-acid substitutions in the spike S1 domain compared with the WT virus. Many key antigenically relevant mutations in the spike NTD (e.g. G142D) and RBD (e.g. T478K and E484A) are shared by both variants [19], which explains why both of them are antigenically distinct from the WT SARS-CoV-2. However, each of these subvariants has unique mutations, deletions or insertions in the S1 domain of the spike protein including in the NTD (residues: 12–306) and RBD (residues: 306–527). Some of these are known to change the antigenicity of NTD [20,21] or RBDs (e.g. BA.1: G446S and G496S; BA.2: R408S) [19,20]. This may suggest that BA.1 and BA.2 may differ in antigenicity from one another as well as from the WT virus and further studies are needed to assess this.

Our findings that BA.2 and BA.1 PRNT_50_ titres are correlated with each other in vaccinated individuals do not prove that BA.1 and BA.2 are antigenically closely related to each other. For that purpose, we need to consider BA.1 and BA.2 PRNT_50_ titres from non-vaccinated individuals infected with each virus or experimental animals infected or vaccinated with each virus. We found that BA.2 infections in unvaccinated individuals resulted in PRNT_50_ titres to BA.2 that were low, but 2–16-fold higher than to BA.1. A recent report using BA.1 infected hamster sera has shown 2.9-fold lower antibody titres to BA.2 compared with BA.1 virus [22]. Another study assessing neutralisation titres in sera from BA.1 infected hamsters showed eightfold lower neutralisation titres to BA.2, though both are antigenically distant from the WT virus [23].

Our findings suggest that Comirnaty elicits higher PRNT titres than CoronaVac. Those with three doses of Comirnaty or convalescent from SARS-CoV-2 with one dose of Comirnaty had PRNT_50_ antibody titres above the predicted protective threshold (titre of 25.6; 95% CI: 18.3–36.0) at 3–5 weeks after the last dose of vaccine. This is concordant with preliminary vaccine effectiveness data against BA.2 that appear to show increased protection after the third dose of an RNA vaccine [10]. Sixteen of 20 individuals receiving two doses of CoronaVac followed by a third dose of Comirnaty had antibody titres above the predicted protective threshold, but GMT titres (46.0; 95% CI: 29.2–72.3) were generally lower than those with three doses of Comirnaty (GMT: 95.1; 95% CI: 73.7–122.8). In contrast, none of 20 people vaccinated with three doses of CoronaVac had PRNT_50_ titres above the predicted protective threshold, even at 3–5 weeks after the last vaccine dose although 15 of them had detectable PRNT_50_ antibody to BA.2. These findings were very similar to what we had previously reported for BA.1 [6].

Our previous studies [6] and those of others have shown that two doses of Comirnaty or CoronaVac vaccines elicited detectable PRNT antibodies to Omicron subvariant BA.1 in only eight of 31 and none of 30 individuals, respectively, and were of low titres even if detectable. Given our current findings suggesting BA.2 PRNT antibody titres are comparable to those of BA.1, this suggests that two doses of these vaccines would also not provide robust protective neutralising antibody to BA.2 subvariant.

A recent large outbreak of BA.2 infection in Hong Kong in January–April 2022 in a population largely naïve to prior natural infection, provided an opportunity to assess the protection provided by Comirnaty and CoronaVac vaccines against mild–moderate disease or severe–fatal disease [24]. While two doses of either vaccine provided minimal protection from mild–moderate infection, the vaccine effectiveness of three doses of Comirnaty was 71.5% (95% CI: 54.5–82.1) and that of three doses of CoronaVac was 42.3% (95% CI: 11.4–62.4), respectively, in those aged 20–59 years. This was concordant with our current observation that Comirnaty vaccination resulted in higher BA.2 PRNT_50_ titres than CoronaVac. Interestingly, vaccine effectiveness against severe disease and death in those given three doses of Comirnaty and CoronaVac vaccines was 98.5% (95% CI: 95.9–99.4) and 98.5% (95% CI: 95.2–99.5), respectively. In the current study, PRNT_50_ antibody to BA.2 was detectable at a dilution of ≥ 10 in all of those given three doses of Comirnaty vaccine and 15 of 20 people vaccinated with three doses of CoronaVac vaccine. Predictive thresholds of neutralising antibody titres for disease severity have also been assessed by Khouri et al., although the confidence CIs are wide [11]. In our PRNT_50_ assay this would be equivalent to a titre of 3.9 (95% CI: 1–17), titres below the resolution of the PRNT_50_ assay. It may be that almost all those given three doses of CoronaVac vaccine did have titres above that range.

Our PRNT assays were done 3–5 weeks after the last vaccine dose. However, a 4.7–6.4-fold waning of neutralising antibody has been reported after two doses of Comirnaty or CoronaVac vaccination by 5 to 6 months after last vaccine dose [25,26]. It is not clear whether the kinetics of antibody waning after a third dose are similar to those after two doses or whether waning of cross-reactive neutralising antibody to variants such as Omicron BA.1 or BA.2 will follow similar kinetics to the WT viruses. It is also relevant to note that preliminary vaccine effectiveness for Omicron subvariant BA.1 does show evidence of waning over time [8].

Although individuals convalescing from WT SARS-CoV-2 had low (GMT: 9.0; 95% CI: 6.7–12.2) BA.2 PRNT_50_ antibody titres, one dose of Comirnaty vaccine was sufficient to elicit markedly higher PRNT_50_ antibodies (GMT: 144.2; 95% CI: 113.2–183.6) than three doses of Comirnaty in infection naïve individuals (GMT: 95.1; 95% CI: 45.5–99.6). These findings are similar to what was observed with BA.1 [6] and suggest that ‘hybrid immunity’ may be superior to immunity from vaccine alone, as regards the neutralising antibody responses [6,27,28]. SARS-CoV-2 convalescent individuals given one dose of CoronaVac also elicited significantly higher PRNT_50_ titres (GMT: 28.3; 95% CI: 20.8–38.4) compared with three doses of CoronaVac vaccine (GMT: 9.3; 95% CI: 7.8–11.5).

In BA.1 and BA.2 breakthrough infections occurring in vaccinated individuals, we saw rapid and high boost in neutralising antibody levels to the infecting virus subvariant as well as broad cross-neutralisation of other variants (Delta and Beta) and WT virus (Table 2, Figure 3). Similar data have been previously reported with COVID-19 Vaccine Janssen immunisations for WT, Beta and Delta variants, and for Omicron [12,27]. Thus, it appears that breakthrough infections in vaccinees lead to eliciting broad cross-neutralisation to a range of virus variants, and one may speculate whether this increased breadth of reactivity may also cover future variants, reflecting a different aspect of ‘hybrid immunity’ [28]. Given the large number of Omicron BA.1 or BA.2 infections that have taken place in countries with relatively high vaccination rates such as United Kingdom [29], it is likely that many of these were vaccine breakthrough infections. Thus, our findings may imply that individuals with breakthrough infections have broad cross-immunity to SARS-CoV-2 variants and this may in turn have positive public health implications at the population level. In marked contrast, in those without prior vaccination or infection, BA.2 infections led to low PRNT_50_ antibody responses to BA.2 and lower cross-reactivity to other variants.

This study has a number of limitations. Only 20 individuals in each vaccine or convalescent group were studied. However, they were randomly drawn from larger cohorts and were likely to be representative. Furthermore, power calculations had suggested that these numbers were more-than-adequate to show statistically significant differences between individual groups, and this is what was observed.

Secondly, the protective correlates we have used primarily relate to protection from symptomatic reinfection, not necessarily severe disease and death. Vaccines, including inactivated vaccines may be more effective at protecting from hospitalisation and severe disease, for example, via T-cell immunity. We have not investigated vaccine-elicited cross-reactive T-cell against BA.2. It is known that T-cell responses can contribute to protection from infection and disease [30] and that T-cell epitopes are substantially conserved between BA.1 and ancestral virus [31]. However, quantitative correlates of T-cell responses that may relate to protection are lacking.

## Conclusions

Three doses of Comirnaty appeared to elicit higher PRNT antibody titres against Omicron subvariant BA.2 than three doses of CoronaVac. A single dose of either vaccine in those with previous SARS-CoV-2 infection elicited higher PRNT antibody responses than even three doses of the respective vaccine in infection naïve individuals. Breakthrough BA.1 or BA.2 infection in previously vaccinated individuals appeared to provide broad cross-neutralisation against a range of SARS-CoV-2 variants of concern. In contrast, BA.2 infection in non-vaccinated individuals provided low PRNT antibody responses to BA.2 with minimal breadth of cross-neutralisation and they may be susceptible to infection with BA.1 or other variants.

## References

[1] World Health Organization (WHO). Classification of Omicron (B.1.1.529): SARS-CoV-2 Variant of Concern. Geneva: WHO; 2021. Available from: https://www.who.int/news/item/26-11-2021-classification-of-omicron-(b.1.1.529)-sars-cov-2-variant-of-concern

[2] Desingu PA Nagarajan K Dhama K . Emergence of Omicron third lineage BA.3 and its importance. J Med Virol. 2022;94(5):1808-10. 10.1002/jmv.27601 35043399 PMC9015590

[3] Lyngse FP Mortensen LH Denwood MJ Christiansen LE Møller CH Skov RL Transmission of SARS-CoV-2 Omicron VOC subvaraints BA.1 and BA.2: Evidence from from Danish households. medRxiv doi: (2022). 10.1101/2021.12.27.21268278

[4] Wolter N Jassat W Walaza S Welch R Moultrie H Groome M Early assessment of the clinical severity of the SARS-CoV-2 omicron variant in South Africa: a data linkage study. Lancet. 2022;399(10323):437-46. 10.1016/S0140-6736(22)00017-4 35065011 PMC8769664

[5] Mallapaty S Callaway E Kozlov M Ledford H Pickrell J Van Noorden R . How COVID vaccines shaped 2021 in eight powerful charts. Nature. 2021;600(7890):580-3. 10.1038/d41586-021-03686-x 34916666

[6] Cheng SMS Mok CKP Leung YWY Ng SS Chan KCK Ko FW Neutralizing antibodies against the SARS-CoV-2 Omicron variant BA.1 following homologous and heterologous CoronaVac or BNT162b2 vaccination. Nat Med. 2022;28(3):486-9. 10.1038/s41591-022-01704-7 35051989 PMC8940714

[7] Pérez-Then E Lucas C Monteiro VS Miric M Brache V Cochon L Neutralizing antibodies against the SARS-CoV-2 Delta and Omicron variants following heterologous CoronaVac plus BNT162b2 booster vaccination. Nat Med. 2022;28(3):481-5. 10.1038/s41591-022-01705-6 35051990 PMC8938264

[8] UK Health Security Agency. *Technical briefing* 34. 14 Jan 2022. Available from: https://assets.publishing.service.gov.uk/government/uploads/system/uploads/attachment_data/file/1050236/technical-briefing-34-14-january-2022.pdf

[9] Waltz E . Does the world need an Omicron vaccine? What researchers say. Nature. 2022;602(7896):192-3. 10.1038/d41586-022-00199-z 35091718

[10] UK Health Security Agency. SARS-CoV-2 variants of concern and variants under investigation in England. *Technical briefing* 35. 28 Jan 2022. Available from: https://assets.publishing.service.gov.uk/government/uploads/system/uploads/attachment_data/file/1050999/Technical-Briefing-35-28January2022.pdf

[11] Khoury DS Cromer D Reynaldi A Schlub TE Wheatley AK Juno JA Neutralizing antibody levels are highly predictive of immune protection from symptomatic SARS-CoV-2 infection. Nat Med. 2021;27(7):1205-11. 10.1038/s41591-021-01377-8 34002089

[12] Keeton R Richardson SI Moyo-Gwete T Hermanus T Tincho MB Benede N Prior infection with SARS-CoV-2 boosts and broadens Ad26.COV2.S immunogenicity in a variant-dependent manner. Cell Host Microbe. 2021;29(11):1611-1619.e5. 10.1016/j.chom.2021.10.003 34688376 PMC8511649

[13] Mok CKP Cohen CA Cheng SMS Chen C Kwok KO Yiu K Comparison of the immunogenicity of BNT162b2 and CoronaVac COVID-19 vaccines in Hong Kong. Respirology. 2022;27(4):301-10. 10.1111/resp.14191 34820940 PMC8934254

[14] Mok CKP Chen C Yiu K Chan TO Lai KC Ling KC A Randomized Clinical Trial Using CoronaVac or BNT162b2 Vaccine as a Third Dose in Adults Vaccinated with Two Doses of CoronaVac. Am J Respir Crit Care Med. 2022;205(7):844-7. 10.1164/rccm.202111-2655LE 35015969 PMC9836218

[15] Matsuyama S Nao N Shirato K Kawase M Saito S Takayama I Enhanced isolation of SARS-CoV-2 by TMPRSS2-expressing cells. Proc Natl Acad Sci USA. 2020;117(13):7001-3. 10.1073/pnas.2002589117 32165541 PMC7132130

[16] Lau EHY Hui DS Tsang OT Chan WH Kwan MY Chiu SS Long-term persistence of SARS-CoV-2 neutralizing antibody responses after infection and estimates of the duration of protection. EClinicalMedicine. 2021;41:101174. 10.1016/j.eclinm.2021.101174 34746725 PMC8556690

[17] Cromer D Steain M Reynaldi A Schlub TE Wheatley AK Juno JA Neutralising antibody titres as predictors of protection against SARS-CoV-2 variants and the impact of boosting: a meta-analysis. Lancet Microbe. 2022;3(1):e52-61. 10.1016/S2666-5247(21)00267-6 34806056 PMC8592563

[18] Iketani S Liu L Guo Y Liu L Chan JF Huang Y Antibody evasion properties of SARS-CoV-2 Omicron sublineages. Nature. 2022; 604(7906):553-6. 10.1038/s41586-022-04594-4 35240676 PMC9021018

[19] McCallum M Czudnochowski N Rosen LE Zepeda SK Bowen JE Walls AC Structural basis of SARS-CoV-2 Omicron immune evasion and receptor engagement. Science. 2022;375(6583):864-8.; Epub ahead of print. 10.1126/science.abn8652 35076256 PMC9427005

[20] Harvey WT Carabelli AM Jackson B Gupta RK Thomson EC Harrison EM COVID-19 Genomics UK (COG-UK) Consortium . SARS-CoV-2 variants, spike mutations and immune escape. Nat Rev Microbiol. 2021;19(7):409-24. 10.1038/s41579-021-00573-0 34075212 PMC8167834

[21] Liu Y Soh WT Kishikawa JI Hirose M Nakayama EE Li S An infectivity-enhancing site on the SARS-CoV-2 spike protein targeted by antibodies. Cell. 2021;184(13):3452-3466.e18. 10.1016/j.cell.2021.05.032 34139176 PMC8142859

[22] Yamasoba D Kimura I Nasser H Morioka Y Nao N Ito J Virological characteristics of SARS-CoV-2 BA.2 variant. 2022. February 15 2022, bioRxiv 2022.02.14.480335; doi: 10.1101/2022.02.14.480335

[23] Mykytyn AZ Rissmann M Kok A Rosu M Schipper D Breugem TI Omicron BA.1 and BA.2 are antigenically distinct SARS-CoV-2 variants. Preprint. bioRxiv. 2022 10.1101/2022.02.23.481644 PMC9273038 35737747

[24] McMenamin ME Nealon J Lin Y Wong JY Cheung JK Lau EHY Vaccine effectiveness of two and three doses of BNT162b2 and CoronaVac against COVID-19 in Hong Kong. Preprint. medRxiv. 10.1016/S1473-3099(22)00345-0 PMC9286709 35850128

[25] Levin EG Lustig Y Cohen C Fluss R Indenbaum V Amit S Waning Immune Humoral Response to BNT162b2 Covid-19 Vaccine over 6 Months. N Engl J Med. 2021;385(24):e84. 10.1056/NEJMoa2114583 34614326 PMC8522797

[26] Zeng G Wu Q Pan H Li M Yang J Wang L Immunogenicity and safety of a third dose of CoronaVac, and immune persistence of a two-dose schedule, in healthy adults: interim results from two single-centre, double-blind, randomised, placebo-controlled phase 2 clinical trials. Lancet Infect Dis. 2022;22(4):483-95. 10.1016/S1473-3099(21)00681-2 34890537 PMC8651254

[27] Wratil PR Stern M Priller A Willmann A Almanzar G Vogel E Three exposures to the spike protein of SARS-CoV-2 by either infection or vaccination elicit superior neutralizing immunity to all variants of concern. Nat Med. 2022;28(3):496-503. 10.1038/s41591-022-01715-4 35090165

[28] Pilz S Theiler-Schwetz V Trummer C Krause R Ioannidis JPA . SARS-CoV-2 reinfections: Overview of efficacy and duration of natural and hybrid immunity. Environ Res. 2022;209:112911. 10.1016/j.envres.2022.112911 35149106 PMC8824301

[29] Gov.UK Coronavirus (COVID-19) in the UK. Updated Wednesday 27 April 2022. [Accessed: 28 Apr 2022]. Available from: https://coronavirus.data.gov.uk/details/cases

[30] Swadling L Diniz MO Schmidt NM Amin OE Chandran A Shaw E COVIDsortium Investigators . Pre-existing polymerase-specific T cells expand in abortive seronegative SARS-CoV-2. Nature. 2022;601(7891):110-7. 10.1038/s41586-021-04186-8 34758478 PMC8732273

[31] Gao Y Cai C Grifoni A Müller TR Niessl J Olofsson A Ancestral SARS-CoV-2-specific T cells cross-recognize the Omicron variant. Nat Med. 2022;28(3):472-6. 10.1038/s41591-022-01700-x 35042228 PMC8938268

